# Exploring tonsil pathology in PTEN hamartoma syndrome: a cohort study

**DOI:** 10.1017/S0022215124000975

**Published:** 2024-11

**Authors:** Lauren M. Cairns, Callum Findlay, Nimeshi Jayakody, Kwamena Amonoo-Kuofi, Nimesh N. Patel, Katherine L. Lachlan

**Affiliations:** 1 Wessex Clinical Genetics Service, University Hospital Southampton NHS Foundation Trust, Southampton, UK; 2 Royal Berkshire Hospital, Royal Berkshire NHS Foundation Trust, Reading, UK; 3 Clinical and Experimental Sciences, Faculty of Medicine, University of Southampton, Southampton, UK; 4 Department of Otolaryngology, Head and Neck Surgery, University Hospital Southampton NHS Foundation Trust, Southampton, UK; 5 Human Genetics and Genomic Medicine, Faculty of Medicine, University of Southampton, Southampton, UK

**Keywords:** tonsil, tonsillectomy, obstructive sleep apnea, otorhinolaryngology, genetics, pediatrics

## Abstract

**Background:**

PTEN hamartoma tumour syndrome (PHTS) comprises a group of genetic disorders with varied clinical presentations, including macrocephaly, developmental delay, and increased cancer susceptibility. Recent reports have highlighted the occurrence of tonsil-related issues in PHTS.

**Methods:**

Clinical data focusing on tonsil-related pathology and tonsillectomy details (indications, histology and post-operative complications) were collected from 53 patients with PHTS.

**Results:**

Tonsil issues affected 58 per cent of the cohort, with 43 per cent requiring tonsillectomy. Primary indications for tonsillectomy included obstructive sleep apnoea (43 per cent), recurrent tonsillitis (17 per cent) and other causes (17 per cent). Tonsil-related problems were observed both before (45 per cent) and after (55 per cent) PHTS. Tonsillectomy with adenoidectomy was the predominant surgical intervention performed (87 per cent), spanning a broad age range (1–27 years old).

**Discussion:**

Our findings highlight the complex nature of PHTS and its association with tonsil-related pathology, demonstrating its relevance for ENT surgeons. Early recognition and intervention are pivotal for managing sleep apnoea and the associated health problems.

## Introduction

PTEN hamartoma tumour syndrome (PHTS) comprises a heterogeneous autosomal dominant condition encompassing distinct clinical entities including Bannayan–Riley–Ruvalcaba syndrome, Cowden syndrome, Lhermitte–Duclos disease, and macrocephaly–autism/developmental delay syndrome. Pathogenic variants in PTEN (Phosphatase and tensin homolog) underlie the multifaceted PTEN hamartoma tumour syndrome.^1–6^ Marked by macrocephaly, developmental delay, mucocutaneous lesions and an increased susceptibility to diverse cancers (breast, endometrial, thyroid, renal and colon), PHTS presents a diagnostic challenge due to its variable and age-related manifestations.^4,7–11^

Case reports have shown evidence that PHTS is associated with tonsillar pathology, in particular early-onset enlargement of tonsillar tissue. Various ages of onset and severity of airway obstruction have been illustrated in the current literature. Mild tonsillar hypertrophy and a papilloma were found in a 2-year-old child, with the papilloma becoming larger and rougher after 6 months.^12^ A 3-year-old boy was noted to have hyperplasia of the pharyngeal tonsils,^13^ while a 6-year-old girl had more severe tonsillar hypertrophy resulting in sleep apnoea and subsequent tonsillectomy^14^ and an 11-year-old boy developed open-mouth breathing from hypertropic tonsils and adenoid vegetation.^15^ Notably, an adult (55-year-old woman) was found to have airway obstruction during induction of general anaesthetic caused by multiple papillomas on the lingual tonsils, epiglottis and the surrounding structure. She was diagnosed with PHTS post operatively.^16^

Despite these case report findings, studies examining tonsillar pathology within a specific PHTS patient cohort are absent. Our case series addresses this gap by reviewing the tonsil-related pathology in this distinct patient population, through rigorous examination of clinical records, diagnostic imaging and histopathological data.

## Methods

Fifty-three patients with PTEN mutations known to the Wessex Clinical Genetics Service were included in the study. The local electronic patient records of these patients were reviewed April–June 2023. Information was extracted including the date and age of PTEN diagnosis, individual's genetic mutation, tonsil pharyngeal conditions, ENT operations, histology and post-operative course, focusing on tonsil issues and subsequent tonsillectomy details.

## Results

Fifty-three patients diagnosed with PHTS were included in this study. Among these patients, 28 (53 per cent) were adults, while 25 (47 per cent) were children. The gender distribution was 33 (62 per cent) male and 20 (38 per cent) female.

Tonsil-related issues were prevalent in 31 (58 per cent) of the PHTS patients, with 23 (74 per cent) of them undergoing subsequent tonsillectomy (43 per cent of the total cohort). The primary indication for tonsillectomy was obstructive sleep apnoea (OSA), accounting for 10 (43 per cent) cases, followed by recurrent tonsillitis in 4 (17 per cent) cases, unknown indications in 4 (17 per cent) cases, and both OSA and recurrent tonsillitis in 3 (13 per cent) cases. Additionally, one patient exhibited asymmetrical tonsils, and another required emergency surgery due to acute airway obstruction.

Eight of the 53 patients (15 per cent) exhibited tonsil issues but did not undergo surgical intervention. These individuals all presented with enlarged tonsils, with five (63 per cent) having normal sleep study results, two displaying possible sleep apnoea and having ongoing surveillance, and one showing no symptoms. Regarding the timing of tonsil issues in relation to PHTS diagnosis, 14 (45 per cent) patients experienced tonsil problems before the PHTS diagnosis, while 17 (55 per cent) developed these issues after the diagnosis.

Tonsillectomy was the predominant surgical procedure, all patients who underwent surgery had this procedure, along with adenoidectomy in 20 (87 per cent) cases. One adenoidectomy was a revision following a previous procedure that involved adenoidectomy and grommet insertion. The age range of PHTS patients undergoing tonsillectomy spanned 1–27 years old, with a mean age of 7.8 years. When the analysis was restricted to only include patients under 30 years of age at the time of data collection, there was an increase in the percentage of tonsil issues (71 per cent, 25/35) and tonsillectomies (49 per cent, 17/35) compared to the whole cohort (58 per cent and 43 per cent, respectively). This reflects an age-related ascertainment bias likely due to older patients not having tonsil-related diagnoses recorded.

No cases were severe enough for a post-op high dependency unit bed. Histology information collection was limited due to surgeries taking place across different hospitals and years (1979–2020). Nonetheless, histology details were accessible for the four (17 per cent) patients who underwent surgery in Southampton within the last decade, all indicating benign reactive lymphoid hyperplasia.

Among the PHTS patients who underwent tonsillectomies, six (26 per cent) experienced post-surgery problems. These complications included ongoing loud snoring but with resolved apnoeas and normal sleep studies, tonsil remnants, hamartomas in the nasopharynx and tongue base, and a single case of recurrent adenoidal hypertrophy necessitating a repeat adenoidectomy six years after the initial procedure.

## Discussion

The present study offers significant insights into the relationship between PTEN Hamartoma Syndrome (PHTS) and tonsil pathology, shedding light on the prevalence, characteristics and clinical implications of tonsil-related pathology within this patient population. Our findings underscore that tonsil-related issues are a common problem in patients with PHTS that should be considered in their medical management.

- PTEN Hamartoma Tumour Syndrome (PHTS) encompasses a group of genetic disorders with varied clinical presentations (macrocephaly, developmental delay, mucocutaneous lesions and increased cancer susceptibility)
- Recent case reports have illustrated the occurrence of tonsil-related issues in PHTS
- Tonsils issues were prevalent in 58% of the cohort
- Forty-three per cent of the PHTS cohort required tonsillectomy, compared to 13.6 per cent of the general population
- This study highlights the association of PHTS with tonsillar pathology throughout early life and its relevance for ENT surgeons
- Early recognition and intervention are pivotal for managing sleep apnoea and associated health problems

Our study cohort consisted of 53 patients diagnosed with PHTS, exhibiting a gender distribution favouring males (62 per cent) and encompassing a diverse age range spanning from children to adults. Tonsil issues emerged as a significant concern within our study cohort, affecting 58 per cent of patients, much greater than the 11 per cent incidence previously reported in school-aged children.^17^ These findings highlight the importance of recognising tonsil pathology as a potential aspect of PHTS that demands clinical attention. Moreover, among those with tonsil-related issues, 74 per cent required tonsillectomy, a surgical intervention to remove the palatine tonsils for histological analysis or remove tissue causing upper airway obstruction. This accounted to 43 per cent of the total cohort which is significantly more than the 13.6 per cent of the general population undergoing tonsillectomy.^18^ The primary indication for tonsillectomy was obstructive sleep apnoea (OSA), a finding consistent with reports of increased OSA prevalence in individuals with PHTS. Importantly, this OSA was never associated with peri-operative respiratory complications so high dependency unit and intensive therapy unit support were not required. Our study further delineated other indications for tonsillectomy, including recurrent tonsillitis. Notably, cases of asymmetrical tonsils and emergency surgical intervention for acute airway obstruction underscore the severe and sometimes urgent nature of tonsil-associated complications in PHTS.

Tonsillectomy was the principal surgical approach, often accompanied by adenoidectomy (87 per cent). The inclusion of adenoidectomy is consistent with the need to address upper airway obstruction and sleep apnoea in individuals with PHTS. The rates of complications and revision surgery were not unexpected and are comparable to adenotonsillectomy in other groups.

Histological evaluation presented challenges due to the variety of hospitals and years during which surgeries were performed. Despite this limitation, histology information for a subset of patients undergoing surgery in Southampton within the last decade indicated benign reactive lymphoid hyperplasia. This finding echoes previous reports of lymphoid hyperplasia associated with PHTS and underlines the need for detailed histopathological assessment to guide clinical decisions.^13^

Our results reveal that tonsil-related problems were identified and treated both before and after the diagnosis of PHTS was made. One patient in our cohort was diagnosed after the ENT surgeon performing the tonsillectomy recognised the macrocephaly and developmental delay and referred the patient to clinical genetics. This highlights the need for ENT surgeons to consider PHTS and be aware of its clinical manifestations when treating children for OSA secondary to tonsil hypertrophy.

Distinguishing patients with PHTS from other patients requires a comprehensive approach that considers clinical, genetic and diagnostic factors. Because PHTS encompasses a diverse array of manifestations, including those affecting the head and neck region, distinguishing features can aid ENT surgeons in identifying individuals with PHTS. Some key considerations for ENT surgeons to differentiate patients with PHTS from others are identified below.

### Clinical phenotype

Clinical phenotype may include macrocephaly, mucocutaneous lesions (trichilemmomas, oral papillomas), developmental delay and specific cancer susceptibilities (breast, thyroid, endometrial). The presence of these features, especially when combined, should raise suspicion for PHTS.

### Family history

Inquiring about a family history of PHTS-related conditions in this autosomal-dominant condition may identify other family members with medical history consistent with PHTS, prompting referral to consider genetic testing. If a pathogenic variant is identified in the family, predictive testing can be offered. This facilitates screening and discussion of risk-reducing surgery, decreasing risk of malignancy.

### Histopathological findings

Skin biopsies of characteristic lesions, such as trichilemmomas and oral papillomas, can provide histopathological evidence supporting the diagnosis of PHTS. Consultation with dermatopathologists can aid in recognising these distinctive features.

### Genetic testing

Genetic testing for mutations in the PTEN gene is a cornerstone in diagnosing PHTS. If clinical suspicion is high, patients should be referred to genetics specialists for appropriate genetic counselling and testing. Detection of pathogenic PTEN variants confirms the diagnosis of PHTS.

In summary, distinguishing patients with PTEN Hamartoma Syndrome from others requires a comprehensive approach that integrates clinical assessment, histopathology and collaboration with specialists from various disciplines. A high index of suspicion, particularly when encountering a constellation of characteristic clinical features, can prompt timely diagnosis and appropriate management of individuals with PHTS.

## Conclusion

In conclusion, our study highlights the crossover between PTEN Hamartoma Syndrome (PHTS) and ENT pathology. By reviewing a cohort of 53 PHTS patients, we found that tonsil hyperplasia is a common condition in patients with PHTS and frequently causes obstructive sleep apnoea in the first decade of life.

The prevalence of tonsil issues in our study cohort emphasises the need for targeted clinical evaluation for this. Clinical geneticists and paediatricians caring for patients with PHTS should consider whether the patient has OSA and examine their tonsils. Conversely, ENT surgeons should consider whether children presenting with OSA and tonsillar hypertrophy also exhibit other signs of PHTS and consider referral for genetic testing.
